# Extraintestinal Manifestations in Inflammatory Bowel Disease: A Retrospective Observational Study in a Peruvian Tertiary Hospital

**DOI:** 10.3390/medicina62081471

**Published:** 2026-07-29

**Authors:** Miguel A. Arce-Huamani, Alvaro N. Sicos-Hancari, Maria D. Vallejo-Jaime, Ayde M. Quispe-Oncebay, Irene Cárdenas-Vela, Jacqueline Abad-Núñez, Luis Cervera-Caballero, Ofelia Castillo-Contreras

**Affiliations:** 1 Research for Evidence, Care, and Public Health, Programa Academico de Medicina Humana, Facultad de Ciencias de la Salud, Universidad Privada Norbert Wiener, Lima 15046, Peru; a2022203014@uwiener.edu.pe (A.N.S.-H.); maria.vallejoj@uwiener.edu.pe (M.D.V.-J.); ayde.quispe@uwiener.edu.pe (A.M.Q.-O.); 2 Hospital Nacional Edgardo Rebagliati Martins, Lima 15046, Peru; ireneesmeralda99@gmail.com (I.C.-V.); jacquiabadnunez@gmail.com (J.A.-N.); cervsteel@gmail.com (L.C.-C.); obcastillocontreras@gmail.com (O.C.-C.)

**Keywords:** inflammatory bowel disease, extraintestinal manifestations, ulcerative colitis, Crohn’s disease, Montreal classification

## Abstract

*Background and Objectives*: Extraintestinal manifestations (EIMs) are clinically relevant components of inflammatory bowel disease (IBD), but real-world data from Latin American referral settings remain limited. This study aimed to estimate the frequency and pattern of documented EIMs and identify associated factors in a Peruvian tertiary-care cohort. *Materials and Methods*: We conducted a retrospective analytical study at the Gastroenterology Service of Hospital Nacional Edgardo Rebagliati Martins, Lima, Peru. Adults with confirmed IBD attended between April 2020 and February 2025 were included (*n* = 162). EIMs were defined as documented articular, dermatologic, or ocular involvement. Crude and adjusted odds ratios (ORs and aORs) with 95% confidence intervals (CIs) were estimated using logistic regression. *Results*: The mean age was 50.6 ± 18.3 years, 54.9% were female, and 71.0% had ulcerative colitis. Overall, 16.7% of patients had at least one documented predefined EIM. Articular involvement was the most frequent manifestation (11.7%), whereas dermatologic and ocular manifestations each accounted for 2.5%. In the exploratory adjusted model, the harmonized Montreal category 3 retained a statistically significant association with documented EIMs (aOR 3.12, 95% CI 1.19–8.18; *p* = 0.021). *Conclusions*: In this Peruvian tertiary IBD cohort, documented EIMs affected a meaningful minority of patients and were predominantly articular. The harmonized Montreal category 3 was associated with documented EIMs and may help flag patients for more targeted systemic and multidisciplinary assessment, although this exploratory finding requires external validation.

## 1. Introduction

Inflammatory bowel disease (IBD), comprising Crohn’s disease (CD) and ulcerative colitis (UC), has become an increasingly relevant global public health problem, with contemporary burden estimates showing a sustained rise in incident cases from 1990 to 2021 despite declining age-standardized mortality and disability-adjusted life-year rates [1]. Recent population-based evidence has shown that the global epidemiology of IBD follows different transition stages across regions, with 522 studies from 82 regions documenting marked variation in incidence and prevalence patterns between 1920 and 2024 [2]. In Latin America, the incidence and prevalence of IBD have increased over recent decades, and several countries are now considered to be in either the acceleration stage or the compounding-prevalence stage of the disease burden [3]. Regional data remain heterogeneous; however, Brazil reported 212,026 patients diagnosed with IBD in 2020, while Córdoba, Argentina, reported an age-adjusted IBD incidence of 3.67 cases per 100,000 person-years and a prevalence of 68.8 cases per 100,000 inhabitants [4]. These changes have occurred in a setting where timely access to diagnosis, specialized monitoring, biologic therapy, and multidisciplinary care remains uneven across Latin American health systems [5].

IBD is a systemic immune-mediated condition and is not limited to intestinal inflammation. Extraintestinal manifestations (EIMs) may involve the musculoskeletal, dermatologic, ocular, hepatobiliary, vascular, renal, pulmonary, and oral systems, and recent reviews indicate that they may affect up to 50% of patients during the disease course [6]. The 2024 ECCO guideline emphasizes that EIMs contribute substantially to morbidity, quality-of-life impairment, diagnostic delay, and therapeutic complexity, particularly when they require coordination between gastroenterology, rheumatology, dermatology, ophthalmology, and hepatology [7]. In a recent systematic review and meta-analysis including 52 studies and 352,454 patients, at least one joint, ocular, or skin EIM was reported in 24% of patients with IBD, 27% of those with UC, and 35% of those with CD [8]. Similarly, the prospective French EXTRA study, conducted in 30 referral centers and including 1971 patients with IBD, found that 27.6% had at least one EIM, with a higher frequency in CD than in UC and multiple EIMs in approximately one fifth of affected patients [9].

Musculoskeletal manifestations are among the most frequent EIMs and include peripheral arthritis, axial spondyloarthritis, sacroiliitis, enthesitis, and inflammatory back pain; recent large-scale association analyses suggest that EIMs are not randomly distributed but cluster according to clinical, demographic, and immune-mediated profiles [10]. Some EIMs, such as peripheral arthritis, erythema nodosum, and episcleritis, may parallel intestinal activity, whereas others, including uveitis, axial spondyloarthritis, and primary sclerosing cholangitis, may follow a course that is partly independent of intestinal inflammation [11]. A 2024 systematic review and meta-analysis from the Eastern Mediterranean region reported arthritis frequencies of 7.1% in UC and 13.5% in CD, skin involvement in 9.9% of patients with IBD, ocular involvement in 7.2%, and primary sclerosing cholangitis in 3.5% of patients with UC and 2.7% of those with CD [12]. Ocular disease is less frequent than articular involvement but can be clinically serious; a recent meta-analysis found that uveitis is one of the most relevant ocular EIMs and is more strongly associated with CD than with UC [13].

Hepatobiliary manifestations deserve particular attention because of their prognostic implications. A systematic review and meta-analysis including 64 studies and 776,700 patients estimated the pooled prevalence of primary sclerosing cholangitis at 2.16% among patients with IBD, with higher estimates in UC than in CD and the highest regional estimates reported in South America [14]. Conversely, among patients with primary sclerosing cholangitis, another meta-analysis found that 71.1% had concomitant IBD and 55.9% had UC, confirming the strong bidirectional relationship between these conditions [15]. EIMs also influence therapeutic decisions because some advanced therapies may improve intestinal inflammation without fully controlling systemic manifestations; a 2024 meta-analysis of 61 studies including 13,806 patients found that new EIMs occurred in 8% of patients receiving advanced therapy, with variable improvement in joint manifestations across biologic classes [16].

Despite these advances, evidence on EIMs in Latin America remains limited and is still concentrated in selected referral centers. A recent Latin American study using clinical and genetic variables to predict EIMs included 414 patients with IBD, most of whom had UC, illustrating the emerging regional interest in identifying high-risk profiles but also the need for broader clinical characterization in real-world cohorts [17]. In Peru, recent tertiary-hospital evidence has focused mainly on clinical characteristics and management of IBD in specific groups, such as older adults, rather than on the frequency and distribution of EIMs across the general adult IBD population [18]. Therefore, updated hospital-based data are needed to describe the local burden of EIMs and to support earlier recognition, multidisciplinary referral, and risk stratification in Peruvian patients with IBD.

The objective of this study was to describe the frequency, distribution, and clinical characteristics of documented predefined articular, dermatologic, and ocular EIMs among patients with inflammatory bowel disease treated at a Peruvian tertiary hospital.

## 2. Materials and Methods

### 2.1. Study Design and Setting

We conducted a retrospective observational analytical study using clinical records to identify factors associated with extraintestinal manifestations (EIMs) among adults with inflammatory bowel disease (IBD). The study was performed at the Gastroenterology Service of Hospital Nacional Edgardo Rebagliati Martins (Lima, Peru), using data obtained from institutional clinical records of patients attended between April 2020 and February 2025 (approved by the Institutional Committee of Ethics and Scientific Integrity of Universidad Privada Norbert Wiener; approve code: CIEIC-UPNW; Exp. No. A0047-2025; approve date: 27 May 2025).

### 2.2. Study Population

The study population comprised adults aged 18 years or older with a confirmed diagnosis of IBD, including ulcerative colitis or Crohn’s disease, based on clinical assessment supported by endoscopic, histologic, and/or imaging criteria. Patients were eligible if their clinical records allowed determination of EIM status and included the clinical and sociodemographic variables required for analysis.

Patients were excluded if their clinical documentation did not allow precise classification of EIM status or if they had concomitant autoimmune or systemic diseases distinct from IBD that could interfere with EIM identification. A census approach was applied, including all eligible patients during the study period.

### 2.3. Variables and Definitions

The primary outcome was the presence of documented predefined EIMs, operationalized as a binary variable: present versus absent. Patients were classified as having documented predefined EIMs when articular, dermatologic, or ocular involvement was explicitly recorded in the medical record. Patients without any of these predefined manifestations were classified as having no EIMs for the comparative and regression analyses. Hepatobiliary manifestations, including primary sclerosing cholangitis, were not incorporated into the primary analytic outcome. Although these conditions are clinically relevant manifestations associated with IBD, they were documented in very few patients and could not be ascertained uniformly from the retrospective records. In particular, the diagnosis of primary sclerosing cholangitis generally requires specific biochemical, cholangiographic, and specialist-supported evaluation, which was not systematically available for all patients. Combining these infrequent and diagnostically heterogeneous conditions with articular, dermatologic, and ocular manifestations could therefore have introduced outcome misclassification and unstable estimates. Their exclusion was an operational decision intended to preserve a consistent primary outcome and should not be interpreted as indicating limited clinical relevance.

For consistency, the term “EIMs” used throughout the Section 3, tables, figures, Section 4, and Section 5 refers exclusively to these documented predefined articular, dermatologic, and ocular manifestations, rather than to the complete extraintestinal spectrum of IBD. The source dataset recorded EIMs as a single mutually exclusive categorical variable representing the main documented manifestation. Therefore, coexistence of articular, dermatologic, and ocular manifestations in the same patient could not be assessed reliably.

The main independent variables were sex, age at diagnosis, IBD type, Montreal location/extent category at diagnosis, prior hospitalization, treatment adherence, current intestinal symptoms at evaluation, and treatment pattern. Sex was categorized as male or female. IBD type was categorized as ulcerative colitis or Crohn’s disease. Age at diagnosis was analyzed as a continuous variable.

Because the conventional Montreal classification uses different location and extent categories for Crohn’s disease and ulcerative colitis, the original disease-specific codes were combined into a four-level institutional variable for the pooled analysis. Category 0 corresponded to L1 Crohn’s disease; category 1 corresponded to L2 Crohn’s disease or E1 ulcerative colitis; category 2 corresponded to L3 Crohn’s disease or E2 ulcerative colitis; and category 3 corresponded to L4 Crohn’s disease or E3 ulcerative colitis. No separate category 4 was retained in the final analytic variable. These harmonized categories were operational groupings and should not be interpreted as conventional Montreal categories or as a strictly ordinal measure of disease severity. For regression analysis, category 3 was compared with categories 0–2 because it showed the clearest descriptive contrast according to EIM status.

Prior hospitalization was initially described in four categories: none, one, two, and three or more. For crude regression analysis, hospitalization history was collapsed as any prior hospitalization versus none to reduce sparse-cell instability. Treatment adherence was categorized as good or poor according to the classification documented by the treating clinical team in the medical record. The retrospective records did not consistently specify whether this classification was based on patient self-report, physician judgment, pharmacy dispensing data, or another formal adherence assessment. Therefore, adherence should be interpreted as a routinely documented clinical variable rather than as a measure obtained using a validated adherence instrument.

Current intestinal symptoms were assessed at the evaluated clinical encounter and harmonized as present or absent. For ulcerative colitis, symptoms included diarrhea, rectal bleeding, tenesmus, abdominal pain, or combinations of these manifestations. For Crohn’s disease, the source variable recorded current symptoms as present or absent without a consistently available symptom-specific breakdown. This variable reflected documented symptoms at the evaluated clinical encounter and was not considered a validated measure of inflammatory disease activity. Validated disease activity indices, including the Mayo score for ulcerative colitis and the Crohn’s Disease Activity Index for Crohn’s disease, were not included because they were not systematically calculated or documented across the retrospective records. Similarly, C-reactive protein and fecal calprotectin measurements were not consistently available for all patients or obtained at a standardized time point corresponding to EIM ascertainment. Therefore, these measures could not be incorporated reliably into the comparative or regression analyses.

Treatment pattern was defined according to the main therapeutic regimen documented at the evaluated clinical encounter. The retrospective records did not consistently provide the exact dates of anti-TNF or azathioprine initiation, treatment modification, or EIM onset. Therefore, it was not possible to reliably determine whether these treatments preceded the development of EIMs or were initiated afterward as part of the management of more complex intestinal or extraintestinal disease. The anti-TNF plus azathioprine category was therefore included only in the crude exploratory analysis because it may reflect disease complexity and confounding by indication. Treatment variables were not included in the adjusted model to reduce overfitting and the risk of collider bias related to disease severity. Smoking status was not included because it was not systematically documented in the available clinical records. Approximate disease duration could be derived from age and age at diagnosis, but exact dates of symptom onset and diagnosis were not uniformly available. Previous surgery was also not incorporated as a pooled covariate because surgical history was recorded using different definitions for Crohn’s disease and ulcerative colitis and could not be harmonized reliably across the full cohort.

### 2.4. Data Collection Procedures

Data were abstracted through a structured review of electronic medical records and complementary clinical documentation. EIMs were identified from diagnoses explicitly documented in the medical record by the treating clinical team or, when available, by a relevant specialist in rheumatology, dermatology, or ophthalmology. Specialist confirmation was not uniformly available and was not required for all cases because of the retrospective design. The research team did not independently diagnose or clinically re-adjudicate EIMs. Records containing only nonspecific symptoms without a documented clinical diagnosis, or insufficient information to classify the manifestation, were not considered positive for EIMs. Investigators used a standardized data collection form to capture sociodemographic variables, IBD phenotype at diagnosis, Montreal location/extent category, prior hospitalizations, treatment regimen, medication adherence, current symptoms, medication-related adverse events, and EIM status. Adherence and current symptom status were extracted as documented by the treating clinical team; the investigators did not retrospectively reclassify adherence using pharmacy records or apply a validated adherence questionnaire.

Because the study relied on routinely documented retrospective data, disease activity was not assessed using a standardized protocol at a uniform time point. Mayo and CDAI scores were inconsistently available, and inflammatory biomarkers such as C-reactive protein and fecal calprotectin had been requested according to clinical need rather than as part of a predefined study schedule. Consequently, isolated values could not be assumed to represent disease activity at the time when an EIM developed or was documented.

Data were entered into a password-protected database using coded identifiers. To minimize transcription error, internal consistency checks were performed, and a subset of records underwent verification by cross-review.

### 2.5. Statistical Analysis

Categorical variables were summarized as frequencies and percentages. Continuous variables were summarized as mean and standard deviation. Descriptive comparisons were made between patients with and without EIMs according to the primary analytic EIM definition. Because of small expected cell counts in several categories, these descriptive comparisons were interpreted cautiously, and formal inference was based primarily on crude and adjusted regression models.

Crude odds ratios (ORs) and 95% confidence intervals (CIs) were estimated using univariable logistic regression with EIM status as the dependent variable. Candidate variables were selected based on clinical relevance, observed descriptive contrasts, and the need to avoid sparse-cell instability. Prior hospitalization was collapsed as any prior hospitalization versus none for crude regression analysis. The anti-TNF plus azathioprine comparison was reported descriptively because treatment pattern may reflect disease complexity rather than causal exposure.

To estimate adjusted associations with EIMs, we fitted a parsimonious multivariable logistic regression model. Because only 27 EIM events were available, the number of predictors was deliberately restricted to four clinically relevant variables: IBD type, age at diagnosis, sex, and harmonized Montreal category 3 versus categories 0–2. This corresponded to approximately 6.8 EIM events per predictor parameter. No automated stepwise selection procedure was used. Treatment variables and other factors with sparse categories were excluded from the adjusted model because their inclusion could have increased model instability and overfitting. The Montreal variable was also collapsed into category 3 versus categories 0–2 to reduce sparse-cell problems. Adjusted ORs with 95% CIs and two-sided *p* values were reported. Given the limited event count, the adjusted model was considered exploratory, and its estimates were interpreted cautiously.

Potential confounders not entered into the adjusted model were assessed according to their availability, comparability across IBD subtypes, temporal interpretability, and potential role in the causal pathway. Smoking status was unavailable, previous surgery was not recorded using a harmonized definition across ulcerative colitis and Crohn’s disease, and current treatment did not provide a reliable measure of cumulative or pre-EIM biologic exposure. Approximate disease duration was not included because the limited number of EIM events required restriction of the model and because it was derived from age-related variables rather than uniformly documented dates of disease onset. Residual confounding by these factors could therefore not be excluded.

All analyses were performed using Stata version 18 (StataCorp LLC, College Station, TX, USA). Statistical significance was defined as a two-sided *p* value < 0.05.

### 2.6. Ethical Considerations

This study followed the principles of the Declaration of Helsinki and was conducted using a retrospective design based exclusively on review of existing clinical records, without direct patient contact or modification of clinical care. The protocol, version 01 dated 27 May 2025, was reviewed and approved by the Institutional Committee of Ethics and Scientific Integrity of Universidad Privada Norbert Wiener (CIEIC-UPNW; Exp. No. A0047-2025).

Given the use of secondary clinical data, confidentiality was protected through anonymization and coding, restricted access to records and databases, and reporting of results only in aggregate form.

## 3. Results

The cohort included 162 adults with IBD. The mean age was 50.6 ± 18.3 years, and the mean age at diagnosis was 41.7 ± 17.5 years. Overall, 54.9% of patients were female, and ulcerative colitis was the most frequent IBD type, accounting for 71.0% of the cohort. At diagnosis, most patients were classified within harmonized Montreal location/extent category 3 (40.7%) or category 2 (34.0%). Nearly two-thirds of patients had no prior hospitalizations (63.6%). Using the primary analytic definition, 83.3% of patients (*n* = 135) had no documented predefined articular, dermatologic, or ocular EIMs, whereas 16.7% (*n* = 27) had at least one documented predefined EIM (Table 1). Medication-related adverse events were uncommon, with 90.7% of patients having no recorded adverse event. Treatment was dominated by anti-TNF monotherapy (50.0%) and 5-ASA only (22.2%). Most patients had good documented treatment adherence (80.9%) and no current intestinal symptoms at the evaluated clinical encounter (67.9%) (Table 2).

The distribution of documented predefined EIMs included in the primary analytic definition is shown in Table 3. Articular involvement was the most frequent manifestation, affecting 11.7% of the total cohort (*n* = 19). Dermatologic and ocular manifestations were less frequent, each accounting for 2.5% of the cohort (*n* = 4 and *n* = 4, respectively). No patient was assigned to more than one EIM category in the source dataset. However, because EIMs were recorded using a single mutually exclusive categorical field, this finding should not be interpreted as evidence that overlapping manifestations were clinically absent.

When stratified by EIM status, patients with and without EIMs had broadly similar mean age (48.7 ± 15.1 vs. 51.0 ± 18.9 years) and age at diagnosis (38.4 ± 11.8 vs. 42.4 ± 18.4 years). The EIM group had a higher proportion of Crohn’s disease than the non-EIM group (40.7% vs. 26.7%). Montreal category 3 was also more frequent among patients with EIMs than among those without EIMs (66.7% vs. 35.6%). Prior hospitalization patterns were broadly comparable between groups. Anti-TNF monotherapy was the most common regimen in both groups, although anti-TNF plus azathioprine was more frequent among patients with EIMs (22.2% vs. 8.9%). Good adherence predominated in both groups, and current symptoms were recorded in 37.0% of patients with EIMs and 31.1% of those without EIMs (Table 4).

In crude logistic regression, harmonized Montreal category 3 was associated with higher odds of documented predefined EIMs compared with categories 0–2 (OR 3.63, 95% CI 1.51–8.69; *p* = 0.005). Crohn’s disease showed higher crude odds of EIMs than ulcerative colitis, although this association did not reach statistical significance (OR 1.89, 95% CI 0.80–4.46; *p* = 0.165). Female sex was not associated with EIMs in crude analysis (OR 0.72, 95% CI 0.31–1.65; *p* = 0.526). Prior hospitalization, poor adherence, and current symptoms were also not significantly associated with EIMs. Anti-TNF plus azathioprine showed a positive but non-significant crude association (OR 2.93, 95% CI 0.99–8.66; *p* = 0.085) (Table 5). This finding was interpreted descriptively because treatment timing in relation to EIM onset could not be established and the observed pattern may reflect disease complexity or treatment initiated after EIM recognition rather than causal exposure.

Given the limited number of EIM events, a parsimonious adjusted logistic regression model was fitted. In this exploratory model, harmonized Montreal category 3 retained a statistically significant adjusted association with documented predefined EIMs (aOR 3.12, 95% CI 1.19–8.18; *p* = 0.021). Crohn’s disease showed a positive but non-significant association (aOR 1.55, 95% CI 0.56–4.31; *p* = 0.400). Age at diagnosis was not independently associated with EIMs (aOR 0.98 per year, 95% CI 0.95–1.01; *p* = 0.187), and female sex was also not associated with EIMs after adjustment (aOR 0.78, 95% CI 0.31–1.91; *p* = 0.585) (Table 6).

**Figure 1:**
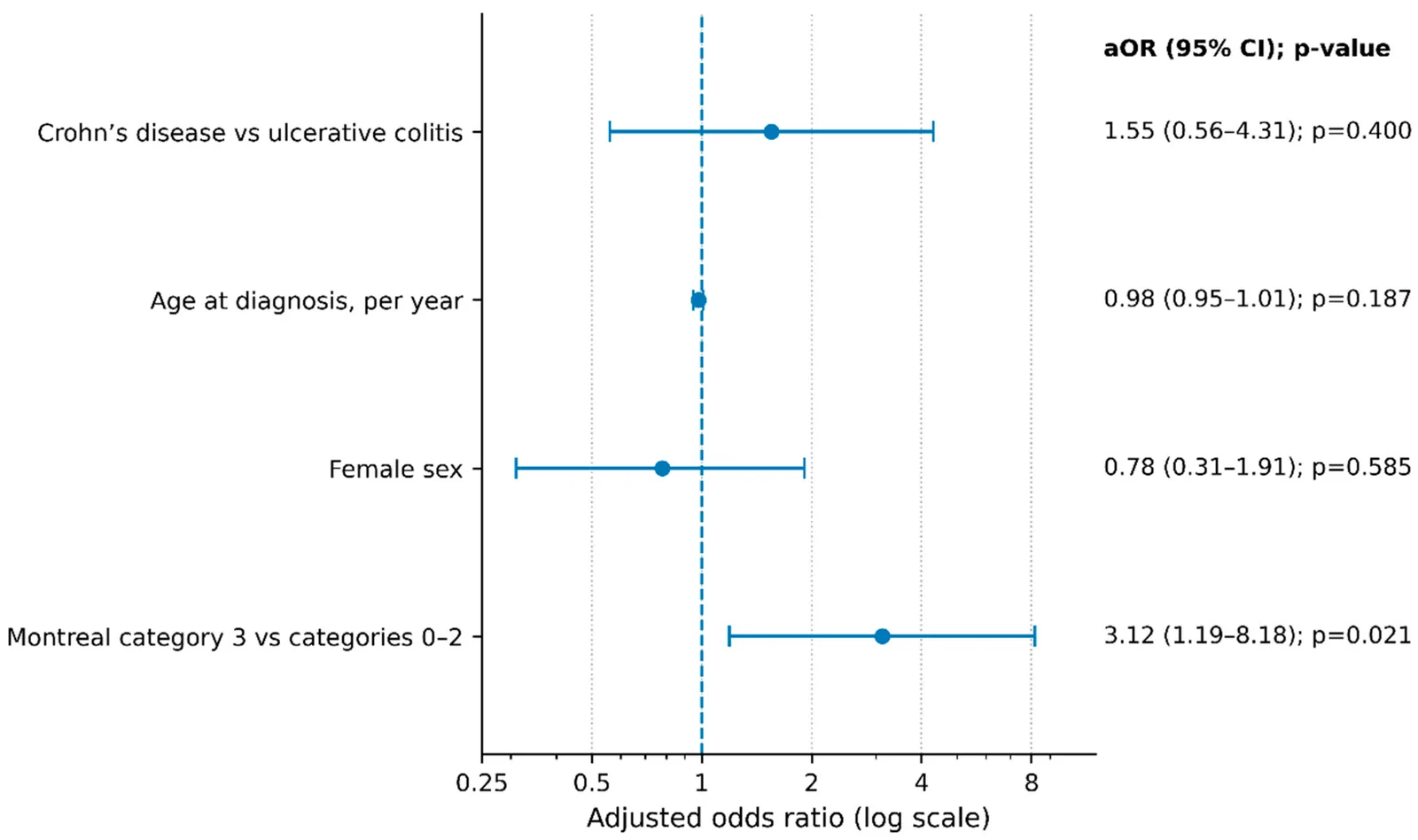
Forest plot of adjusted odds ratios for factors associated with documented predefined extraintestinal manifestations in patients with inflammatory bowel disease. The adjusted model was specified parsimoniously because only 27 EIM events were available. Odds ratios are shown on a logarithmic scale, and horizontal lines represent 95% confidence intervals. Harmonized Montreal category 3 retained a statistically significant adjusted association with EIMs, whereas the confidence intervals for Crohn’s disease, age at diagnosis, and female sex crossed the null value. Given the limited number of EIM events, these estimates should be interpreted as exploratory.

The forest plot shows that harmonized Montreal category 3 was the only variable that retained a statistically significant adjusted association with EIMs in the parsimonious exploratory model, whereas the confidence intervals for Crohn’s disease, age at diagnosis, and female sex crossed the null value (Figure 1).

## 4. Discussion

In this retrospective cohort of adults with inflammatory bowel disease treated at a Peruvian tertiary hospital, nearly one in six patients had at least one documented predefined extraintestinal manifestation involving the articular, dermatologic, or ocular systems. Articular involvement was the predominant manifestation, whereas dermatologic and ocular manifestations were uncommon and no overlap between EIM categories was recorded. Although patients with EIMs showed a higher proportion of Crohn’s disease and more frequent harmonized Montreal category 3 involvement at diagnosis, Montreal category 3 was the only variable that retained a statistically significant adjusted association in the parsimonious exploratory model. These findings suggest that, in this hospital-based cohort, the harmonized category 3 phenotype, comprising E3 ulcerative colitis or L4 Crohn’s disease, was the main clinical factor associated with extraintestinal disease expression.

The frequency of documented predefined EIMs in our cohort was lower than most contemporary international estimates. We identified at least one articular, dermatologic, or ocular EIM in 16.7% of patients. In comparison, Kilic et al. [8] reported these manifestations in 24% of patients with IBD, while the prospective EXTRA study found a prevalence of 27.6% [9]. Khrom et al. [10] and Pérez-Jeldres et al. [17] reported frequencies of approximately 27% and 29%, respectively. Conversely, our estimate was similar to the 16.3% reported by Sîngeap et al. [19]. These differences likely reflect variation in case definitions and ascertainment methods. Because our primary outcome was restricted to manifestations explicitly documented in clinical records and excluded hepatobiliary and other systemic conditions, the observed frequency should not be interpreted as the full extraintestinal burden of IBD.

The exclusion of hepatobiliary manifestations, particularly primary sclerosing cholangitis, further narrowed the primary outcome. Although primary sclerosing cholangitis is a clinically important IBD-associated condition that may evolve independently of intestinal activity [7,14,15], its retrospective identification requires adequate biochemical, imaging, and specialist documentation. These data were not consistently available, and only a few hepatobiliary manifestations were recorded. Their inclusion could therefore have increased diagnostic heterogeneity and produced unstable estimates. Accordingly, the reported prevalence represents documented predefined articular, dermatologic, and ocular EIMs rather than the complete extraintestinal spectrum of IBD.

Retrospective ascertainment may also have contributed to the relatively low frequency observed in our cohort. Only explicitly documented diagnoses were counted; therefore, mild, intermittent, resolved, externally managed, or subclinical manifestations may have been missed. The absence of systematic rheumatologic, dermatologic, and ophthalmologic screening, together with variation in documentation practices, may have further reduced case detection. Accordingly, under-ascertainment may have contributed substantially to the lower frequency compared with prospective studies, and the observed 16.7% should be interpreted as the frequency of documented predefined EIMs rather than the cumulative burden of extraintestinal disease.

The distribution of documented predefined EIMs was consistent with previous evidence. Articular involvement was the most frequent manifestation, whereas dermatologic and ocular involvement was less common. This pattern agrees with Sanat et al. [12], the EXTRA study [9], and the multicohort analysis by Khrom et al. [10], all of which identified musculoskeletal manifestations as the predominant EIM category. ECCO guidelines similarly emphasize the importance of rheumatologic assessment when axial or peripheral involvement is suspected [7]. Although this internal distribution supports the clinical coherence of our findings, the low frequencies of dermatologic and ocular manifestations may partly reflect limited specialist referral, incomplete documentation, and the absence of systematic screening.

No overlap between articular, dermatologic, and ocular EIM categories was identified in the analytic dataset. However, overlapping manifestations are clinically possible in IBD, and this result should be interpreted cautiously. Because the source variable assigned each patient to a single main documented EIM category, coexisting manifestations may have been incompletely recorded or reduced to the manifestation considered most clinically relevant at the evaluated encounter. Therefore, the absence of recorded overlap may reflect the structure of the retrospective classification rather than the true absence of multiple EIMs in individual patients.

Harmonized Montreal category 3 retained a statistically significant adjusted association with documented predefined EIMs in the exploratory model. This operational category combined E3 extensive colitis in ulcerative colitis with L4 upper gastrointestinal involvement in Crohn’s disease. Because these are clinically distinct phenotypes, the combined category should not be interpreted as a conventional Montreal classification or as an ordinal measure of disease severity. Previous studies have reported associations between EIMs and extensive colitis in ulcerative colitis, as well as non-isolated ileal involvement in Crohn’s disease [10,19]. ECCO also recognizes that some EIMs parallel intestinal inflammatory activity, whereas others may evolve independently [7]. Therefore, category 3 may serve as a pragmatic marker for closer systemic assessment, but this exploratory finding requires confirmation in larger disease-specific analyses.

Clinically, harmonized Montreal category 3 may help identify patients who warrant closer assessment for documented predefined EIMs. Routine follow-up could include targeted questioning about inflammatory joint symptoms, skin lesions, and ocular complaints, together with timely referral to rheumatology, dermatology, or ophthalmology when indicated [7]. However, this exploratory category should not be used alone to intensify treatment, determine prognosis, or establish a fixed screening schedule. Rather, it should complement disease activity assessment, treatment history, and individualized clinical judgment.

Crohn’s disease was more frequent among patients with documented predefined EIMs, but the adjusted association was not statistically significant. The direction of the estimate was consistent with previous evidence showing higher frequencies of joint, skin, and ocular manifestations in Crohn’s disease than in ulcerative colitis [8,19]. Pérez-Jeldres et al. [17] reported a similar pattern in a Latin American cohort, while ocular involvement has also been described more frequently in Crohn’s disease [8,20]. However, the wide confidence interval in our analysis indicates limited precision. Therefore, the result should not be interpreted as evidence that Crohn’s disease is unrelated to EIMs, but rather as an inconclusive estimate constrained by the small number of events.

Female sex, age at diagnosis, previous hospitalization, treatment adherence, and current intestinal symptoms were not significantly associated with documented predefined EIMs. However, current symptoms were derived from routine clinical documentation and did not represent a validated measure of disease activity. Mayo and CDAI scores were not systematically available, while C-reactive protein and fecal calprotectin were inconsistently measured in relation to EIM onset. Previous evidence indicates that some EIMs may parallel intestinal activity, whereas others can persist or develop independently [7,9]. Therefore, the absence of an association with current symptoms should not be interpreted as evidence that intestinal inflammation is unrelated to EIMs, and residual confounding by disease activity remains possible.

The crude association between anti-TNF plus azathioprine and documented predefined EIMs should be interpreted cautiously. The retrospective records did not consistently establish whether treatment began before or after EIM onset; therefore, the finding cannot support a causal interpretation. More plausibly, it may reflect confounding by indication, because patients with more complex, refractory, or extraintestinal disease may be more likely to receive combination therapy. Similar treatment patterns among patients with EIMs have been reported previously [17], and responses of EIMs to advanced therapies vary by manifestation and therapeutic agent [16]. Accordingly, this association was retained only as a descriptive crude finding, and longitudinal studies with clearly documented treatment and EIM dates are needed to establish temporality.

The main contribution of this study is the provision of real-world data on documented predefined EIMs in a Peruvian tertiary-care cohort. Articular involvement predominated, and harmonized Montreal category 3 retained a statistically significant adjusted association with EIMs in the exploratory model. However, the single-center referral setting may have selected patients with greater disease complexity, more frequent specialist follow-up, or greater access to advanced therapies. Differences in referral pathways, diagnostic practices, treatment access, and clinical documentation may therefore limit applicability to the broader Peruvian IBD population, rural or non-referral settings, and other healthcare systems. External validation is required before broader application of these findings.

This study has several strengths. It evaluated a clinically characterized cohort of adults with IBD and included relevant demographic, disease-related, treatment, adherence, and hospitalization variables. The analysis used a predefined outcome restricted to documented articular, dermatologic, and ocular EIMs, thereby reducing heterogeneity from less consistently recorded systemic conditions. In addition, the parsimonious multivariable model allowed adjusted associations to be explored while limiting the number of predictors in view of the small number of EIM events.

Several limitations should be acknowledged. The retrospective, single-center design limits causal inference and external validity. The cohort may not represent patients with milder disease managed outside tertiary referral hospitals, and differences in referral criteria, access to biologic therapy, multidisciplinary assessment, and documentation practices may limit applicability to other settings. EIM ascertainment depended on diagnoses recorded in clinical records, without uniform specialist confirmation or independent adjudication. Consequently, mild, intermittent, nonspecific, externally managed, or previously resolved manifestations may have been missed or misclassified, particularly in patients who did not undergo systematic rheumatologic, dermatologic, or ophthalmologic assessment. The reported frequency should therefore be interpreted as the prevalence of documented predefined EIMs rather than the complete cumulative burden of extraintestinal disease.

In addition, EIMs were recorded as a single mutually exclusive category, which prevented reliable assessment of overlapping articular, dermatologic, and ocular manifestations and may have underestimated the complexity of extraintestinal involvement. Treatment adherence was based on the good/poor classification available in routine clinical documentation, but the underlying assessment method was not uniformly recorded. Therefore, potential differences between self-reported adherence, physician assessment, and pharmacy-based adherence could not be evaluated. Current symptom status was also derived from routine documentation and harmonized across IBD subtypes, which may have reduced the clinical detail and comparability of this variable.

Objective disease activity could not be evaluated adequately because Mayo and CDAI scores were not systematically documented, while C-reactive protein and fecal calprotectin were unavailable for many patients or were measured at time points unrelated to EIM ascertainment. Consequently, adjustment for inflammatory activity was not possible, and residual confounding remains likely. Smoking status was also inconsistently documented, while previous surgery could not be harmonized across ulcerative colitis and Crohn’s disease. Approximate disease duration was excluded because disease onset was not uniformly recorded and the limited number of EIM events restricted model complexity. Finally, treatment data did not capture cumulative anti-TNF or azathioprine exposure or consistently establish whether therapy preceded EIM onset. Therefore, confounding by indication and reverse temporality cannot be excluded.

The small number of EIM events reduced statistical power and restricted the complexity of the adjusted model. Although only four clinically relevant predictors were included, the model had approximately 6.8 events per parameter; therefore, some overfitting and coefficient instability cannot be excluded. The adjusted estimates, particularly those with wide confidence intervals, should be interpreted as exploratory. In addition, the cross-sectional assessment prevented evaluation of temporal relationships among intestinal activity, treatment exposure, and EIM onset or remission.

## 5. Conclusions

In conclusion, documented articular, dermatologic, or ocular extraintestinal manifestations were present in a minority of adults with inflammatory bowel disease, with articular involvement being the most frequent manifestation. The harmonized Montreal category 3 retained a statistically significant adjusted association with documented EIMs in the exploratory model, suggesting that this combined phenotype may help flag patients for closer systemic assessment. Although Crohn’s disease and combination therapy with anti-TNF plus azathioprine showed clinically relevant patterns, these findings should be interpreted cautiously because of limited precision and potential confounding by disease severity. Overall, the harmonized category 3 phenotype may serve as a pragmatic clinical alert for targeted review of articular, dermatologic, and ocular symptoms and timely multidisciplinary referral when indicated. Nevertheless, it should not be used as a stand-alone risk-stratification or treatment decision tool, and external validation is required before this association can be applied beyond similar tertiary referral settings.

## Figures and Tables

**Table 1:** Demographic and clinical characteristics of the inflammatory bowel disease cohort.

Variable	*n* (%)
Age, years	50.6 ± 18.3
Age at diagnosis, years	41.7 ± 17.5
Sex	
Male	73 (45.1)
Female	89 (54.9)
IBD type	
Ulcerative colitis	115 (71.0)
Crohn’s disease	47 (29.0)
Harmonized Montreal location/extent category at diagnosis	
Category 0	14 (8.6)
Category 1	27 (16.7)
Category 2	55 (34.0)
Category 3	66 (40.7)
Number of hospitalizations	
None	103 (63.6)
One	38 (23.5)
Two	12 (7.4)
Three or more	9 (5.6)
Documented predefined EIM status	
No articular, dermatologic, or ocular EIM	135 (83.3)
At least one articular, dermatologic, or ocular EIM	27 (16.7)

Note: Data are presented as mean ± standard deviation for continuous variables and *n* (%) for categorical variables. Documented predefined EIMs included articular, dermatologic, or ocular involvement explicitly recorded in the clinical record. Hepatobiliary and other autoimmune manifestations were not included in the primary analytic outcome because of their very low frequency and diagnostic heterogeneity. The Montreal categories were harmonized for the pooled analysis as follows: category 0, L1 Crohn’s disease; category 1, L2 Crohn’s disease or E1 ulcerative colitis; category 2, L3 Crohn’s disease or E2 ulcerative colitis; and category 3, L4 Crohn’s disease or E3 ulcerative colitis. These operational categories do not represent the conventional Montreal classification as a single ordinal scale. Abbreviations: IBD, inflammatory bowel disease; EIM, extraintestinal manifestation.

**Table 2:** Treatment, medication-related adverse events, adherence, and current symptoms.

Variable	*n* (%)
Medication-related adverse events	
None	147 (90.7)
Mild hypersensitivity	5 (3.1)
Severe hypersensitivity	2 (1.2)
Hematologic	2 (1.2)
Dermatologic	2 (1.2)
Cancer	1 (0.6)
Infections, non-TB	1 (0.6)
Tuberculosis	2 (1.2)
Standard treatment, harmonized	
None	7 (4.3)
5-ASA only	36 (22.2)
Azathioprine only	4 (2.5)
Anti-TNF only	81 (50.0)
Ustekinumab	8 (4.9)
Anti-TNF + AZA	18 (11.1)
Anti-TNF + 5-ASA	3 (1.9)
5-ASA + AZA	2 (1.2)
Post-surgery, UC only	3 (1.9)
Treatment adherence	
Good	131 (80.9)
Poor	31 (19.1)
Current intestinal symptoms	
No	110 (67.9)
Yes	52 (32.1)

Note: Data are presented as *n* (%). Medication-related adverse events correspond to events documented in the clinical record during follow-up. Standard treatment reflects the main therapeutic regimen documented at the evaluated clinical encounter. Treatment adherence reflects the good/poor classification recorded by the treating clinical team; the underlying assessment method was not uniformly specified. Current intestinal symptoms were harmonized as present or absent at the evaluated clinical encounter and should not be interpreted as a validated measure of inflammatory disease activity. Abbreviations: UC, ulcerative colitis; TB, tuberculosis; 5-ASA, 5-aminosalicylates; AZA, azathioprine; anti-TNF, anti-tumor necrosis factor.

**Table 3:** Distribution of documented predefined extraintestinal manifestations included in the primary analytic definition.

Type of Extraintestinal Manifestation	*n*	%
None	135	83.3
Articular	19	11.7
Dermatologic	4	2.5
Ocular	4	2.5
Total	162	100.0

Note: This table summarizes the documented predefined EIM categories included in the primary analytic classification. Patients were classified as having EIMs when articular, dermatologic, or ocular involvement was documented in the clinical record. The source variable was mutually exclusive and recorded one main EIM category per patient; therefore, the coexistence of multiple EIM categories could not be evaluated reliably. Abbreviations: EIM, extraintestinal manifestation.

**Table 4:** Descriptive characteristics according to the presence of documented predefined extraintestinal manifestations.

Variable	No EIMs (*n* = 135)	With EIMs (*n* = 27)
Age, years	51.0 ± 18.9	48.7 ± 15.1
Age at diagnosis, years	42.4 ± 18.4	38.4 ± 11.8
IBD type		
Ulcerative colitis	99 (73.3)	16 (59.3)
Crohn’s disease	36 (26.7)	11 (40.7)
Sex		
Male	59 (43.7)	14 (51.9)
Female	76 (56.3)	13 (48.1)
Harmonized Montreal location/extent category at diagnosis		
Category 0	12 (8.9)	2 (7.4)
Category 1	24 (17.8)	3 (11.1)
Category 2	51 (37.8)	4 (14.8)
Category 3	48 (35.6)	18 (66.7)
Number of hospitalizations		
0	88 (65.2)	15 (55.6)
1	30 (22.2)	8 (29.6)
2	9 (6.7)	3 (11.1)
≥3	8 (5.9)	1 (3.7)
Standard treatment		
None	7 (5.2)	0 (0.0)
5-ASA only	33 (24.4)	3 (11.1)
Azathioprine only	4 (3.0)	0 (0.0)
Anti-TNF only	66 (48.9)	15 (55.6)
Ustekinumab	8 (5.9)	0 (0.0)
Anti-TNF + AZA	12 (8.9)	6 (22.2)
Anti-TNF + 5-ASA	2 (1.5)	1 (3.7)
5-ASA + AZA	2 (1.5)	0 (0.0)
Post-surgery	2 (1.5)	1 (3.7)
Treatment adherence		
Good	111 (82.2)	20 (74.1)
Poor	24 (17.8)	7 (25.9)
Current symptoms		
No	93 (68.9)	17 (63.0)
Yes	42 (31.1)	10 (37.0)

**Note:** Data are presented as mean ± standard deviation for continuous variables and *n* (%) for categorical variables. EIMs were defined according to the primary analytic classification, including articular, dermatologic, or ocular involvement. Because of small expected cell counts in several categories, this table is presented descriptively and formal inference is reported in the crude and adjusted regression models. Treatment adherence reflects the classification documented in the clinical record, whereas current symptoms were harmonized as present or absent at the evaluated clinical encounter. Neither variable was obtained using a standardized validated instrument. The Montreal categories were harmonized for the pooled analysis as follows: category 0, L1 Crohn’s disease; category 1, L2 Crohn’s disease or E1 ulcerative colitis; category 2, L3 Crohn’s disease or E2 ulcerative colitis; and category 3, L4 Crohn’s disease or E3 ulcerative colitis. These operational categories do not represent the conventional Montreal classification as a single ordinal scale. Abbreviations: EIM, extraintestinal manifestation; IBD, inflammatory bowel disease; 5-ASA, 5-aminosalicylates; AZA, azathioprine; anti-TNF, anti-tumor necrosis factor.

**Table 5:** Crude odds ratios for factors associated with documented predefined extraintestinal manifestations in patients with inflammatory bowel disease.

Variable	Crude OR	95% CI	*p* Value
Female sex	0.72	0.31–1.65	0.526
Crohn’s disease vs. ulcerative colitis	1.89	0.80–4.46	0.165
Montreal category 3 vs. categories 0–2	3.63	1.51–8.69	0.005
Any prior hospitalization vs. none	1.50	0.65–3.46	0.384
Poor adherence vs. good adherence	1.62	0.62–4.26	0.420
Current symptoms vs. no symptoms	1.30	0.55–3.08	0.652
Anti-TNF + AZA vs. other treatment regimens	2.93	0.99–8.66	0.085

Note: Crude odds ratios were estimated using the primary analytic EIM definition as the dependent variable. Prior hospitalization was collapsed as any prior hospitalization versus none to reduce sparse-cell instability. The anti-TNF + AZA comparison is reported descriptively because the available records did not consistently establish whether treatment preceded or followed EIM onset, and the observed association may reflect disease complexity or confounding by indication rather than causal exposure. Abbreviations: OR, odds ratio; CI, confidence interval; EIM, extraintestinal manifestation; AZA, azathioprine; anti-TNF, anti-tumor necrosis factor.

**Table 6:** Parsimonious adjusted model for factors associated with documented predefined extraintestinal manifestations in patients with inflammatory bowel disease.

Variable	Adjusted OR	95% CI	*p* Value
Crohn’s disease vs. ulcerative colitis	1.55	0.56–4.31	0.400
Age at diagnosis, per year	0.98	0.95–1.01	0.187
Female sex	0.78	0.31–1.91	0.585
Montreal category 3 vs. categories 0–2	3.12	1.19–8.18	0.021

Note: This parsimonious adjusted model was specified because only 27 EIM events were available. The model included four predictor parameters, corresponding to approximately 6.8 EIM events per parameter. Predictor inclusion was restricted based on clinical relevance and the need to reduce overfitting and sparse-cell instability; no automated stepwise selection procedure was used. Age at diagnosis was modeled as a continuous variable. Harmonized Montreal category 3 was compared against categories 0–2. The reference categories were ulcerative colitis, male sex, and Montreal categories 0–2. Treatment variables were not included because of the limited event count, sparse categories, and the possibility of confounding by indication. Therefore, the adjusted estimates should be interpreted as exploratory. *p* values are two-sided. Abbreviations: OR, odds ratio; CI, confidence interval; EIM, extraintestinal manifestation.

## Data Availability

The de-identified dataset and analytic syntax are available from the corresponding author upon reasonable request, subject to institutional approval and applicable ethical restrictions.

## Abbreviations

The following abbreviations are used in this manuscript:

IBD	Inflammatory bowel disease
UC	Ulcerative colitis
CD	Crohn’s disease
EIMs	Extraintestinal manifestations
OR	Odds ratio
aOR	Adjusted odds ratio
CI	Confidence interval
SD	Standard deviation
PSC	Primary sclerosing cholangitis
TB	Tuberculosis
5-ASA	5-aminosalicylates
AZA	Azathioprine
anti-TNF	Anti–tumor necrosis factor
CIEIC-UPNW	Institutional Committee of Ethics and Scientific Integrity of Universidad Privada Norbert Wiener

## References

[1] Lin D., Jin Y., Shao X., Xu Y., Ma G., Jiang Y., Xu Y., Jiang Y., Hu D. (2024). Global, Regional, and National Burden of Inflammatory Bowel Disease, 1990–2021: Insights from the Global Burden of Disease 2021. Int. J. Color. Dis..

[2] Hracs L., Windsor J.W., Gorospe J., Cummings M., Coward S., Buie M.J., Quan J., Goddard Q., Caplan L., Markovinović A. (2025). Global Evolution of Inflammatory Bowel Disease across Epidemiologic Stages. Nature.

[3] Balderramo D., Quaresma A.B., Olivera P.A., Savio M.C., Villamil M.P.G., Panaccione R., Ng S.C., Kaplan G.G., Kotze P.G. (2024). Challenges in the Diagnosis and Treatment of Inflammatory Bowel Disease in Latin America. Lancet Gastroenterol. Hepatol..

[4] Queiroz N.S.F., de Martins C.A., Quaresma A.B., Olivera Sendra P.A., Ernest-Suarez K., Kotze P.G. (2023). IBD Barriers across the Continents: A Continent-Specific Analysis: Latin America. Ther. Adv. Gastroenterol..

[5] Steinwurz F., Machado M.B., Veitia G., De Paula J.A., Bautista Martinez S., Vergara B.I., Capdevielle B., Martinez Silva F.A., Ramirez A.L. (2023). Latin America Consensus Statement Inflammatory Bowel Disease: Importance of Timely Access to Diagnosis and Treatment. Ther. Adv. Gastroenterol..

[6] Faggiani I., Fanizza J., D’Amico F., Allocca M., Zilli A., Parigi T.L., Barchi A., Danese S., Furfaro F. (2024). Extraintestinal Manifestations in Inflammatory Bowel Disease: From Pathophysiology to Treatment. Biomedicines.

[7] Gordon H., Burisch J., Ellul P., Karmiris K., Katsanos K., Allocca M., Bamias G., Barreiro-de Acosta M., Braithwaite T., Greuter T. (2024). ECCO Guidelines on Extraintestinal Manifestations in Inflammatory Bowel Disease. J. Crohns Colitis.

[8] Kilic Y., Kamal S., Jaffar F., Sriranganathan D., Quraishi M.N., Segal J.P. (2024). Prevalence of Extraintestinal Manifestations in Inflammatory Bowel Disease: A Systematic Review and Meta-Analysis. Inflamm. Bowel Dis..

[9] Guillo L., Savoye G., Amiot A., Gilletta C., Nachury M., Dib N., Bourreille A., Roblin X., Caillo L., Allez M. (2023). Prevalence of and Factors Associated with Extraintestinal Manifestations and Their Remission in Inflammatory Bowel Disease: The EXTRA-Intestinal Manifestation Prospective Study from the Groupe d’Etude Thérapeutique des Affections Inflammatoires Du Tube Digestif. Clin. Transl. Gastroenterol..

[10] Khrom M., Long M., Dube S., Robbins L., Botwin G.J., Yang S., Mengesha E., Li D., Naito T., Bonthala N.N. (2024). Comprehensive Association Analyses of Extraintestinal Manifestations in Inflammatory Bowel Disease. Gastroenterology.

[11] Rogler G., Singh A., Kavanaugh A., Rubin D.T. (2021). Extraintestinal Manifestations of Inflammatory Bowel Disease: Current Concepts, Treatment, and Implications for Disease Management. Gastroenterology.

[12] Momayez Sanat Z., Vahedi H., Malekzadeh R., Fanni Z. (2024). A Systematic Review and Meta-Analysis of Extra-Intestinal Manifestation of Inflammatory Bowel Disease in the Eastern Mediterranean Region (EMRO) Countries. Ann. Med. Surg..

[13] Li J.-X., Chiang C.-C., Chen S.-N., Lin J.-M., Tsai Y.-Y. (2022). The Prevalence of Ocular Extra-Intestinal Manifestations in Adults Inflammatory Bowel Disease: A Systematic Review and Meta-Analysis. Int. J. Environ. Res. Public Health.

[14] Barberio B., Massimi D., Cazzagon N., Zingone F., Ford A.C., Savarino E.V. (2021). Prevalence of Primary Sclerosing Cholangitis in Patients with Inflammatory Bowel Disease: A Systematic Review and Meta-Analysis. Gastroenterology.

[15] Zhang Y., Gao X., He Z., Jia H., Chen M., Wang X., Hong L., Cui Y., Wan J. (2022). Prevalence of Inflammatory Bowel Disease in Patients with Primary Sclerosing Cholangitis: A Systematic Review and Meta-Analysis. Liver Int. Off. J. Int. Assoc. Study Liver.

[16] Tímár Á.E., Párniczky A., Budai K.A., Hernádfői M.V., Kasznár E., Varga P., Hegyi P., Váncsa S., Tóth R., Veres D.S. (2024). Beyond the Gut: A Systematic Review and Meta-Analysis of Advanced Therapies for Inflammatory Bowel Disease-Associated Extraintestinal Manifestations. J. Crohn’s Colitis.

[17] Pérez-Jeldres T., Reyes-Pérez P., Gonzalez-Hormazabal P., Avendano C., Segovia Melero R., Azocar L., Silva V., De La Vega A., Arriagada E., Hernandez E. (2025). Prediction of Extraintestinal Manifestations in Inflammatory Bowel Disease Using Clinical and Genetic Variables with Machine Learning in a Latin IBD Group. Int. J. Mol. Sci..

[18] Méndez J.E.P., Junes S.I.P., Marcacuzco H.T.V., Méndez E.M.V. (2023). Enfermedad Inflamatoria Intestinal en el adulto mayor: Características clínicas y manejo en un hospital de referencia. Rev. Gastroenterol. Perú.

[19] Singeap A.-M., Girleanu I., Diculescu M., Gheorghe L., Ciocîrlan M., Gheorghe C., Costache A., Tanțău A., Zaharie R., Goldis A. (2021). Risk Factors for Extraintestinal Manifestations in Inflammatory Bowel Diseases—Data from the Romanian National Registry. J. Gastrointest. Liver Dis. JGLD.

[20] Shah J., Shah A., Hassman L., Gutierrez A. (2021). Ocular Manifestations of Inflammatory Bowel Disease. Inflamm. Bowel Dis..

